# Characteristics of Rhizosphere Microbiome, Soil Chemical Properties, and Plant Biomass and Nutrients in *Citrus reticulata* cv. Shatangju Exposed to Increasing Soil Cu Levels

**DOI:** 10.3390/plants13172344

**Published:** 2024-08-23

**Authors:** Xiaorong Mo, Qichun Huang, Chuanwu Chen, Hao Xia, Muhammad Riaz, Xiaomin Liang, Jinye Li, Yilin Chen, Qiling Tan, Songwei Wu, Chengxiao Hu

**Affiliations:** 1 Guangxi Key Laboratory of Marine Environment Change and Disaster in Beibu Gulf, College of Resources and Environment, Beibu Gulf University, Qinzhou 535011, China; xrmo@bbgu.edu.cn; 2 Microelement Research Center, Hubei Provincial Engineering Laboratory for New Fertilizers, Key Laboratory of Arable Land Conservation (Middle and Lower Reaches of Yangtze River), Ministry of Agriculture, Huazhong Agricultural University, Wuhan 430070, China; ami113@foxmail.com (X.L.); 19906404893@163.com (J.L.); whchenyilin10@gmail.com (Y.C.); qltan@mail.hzau.edu.cn (Q.T.); wusw@mail.hzau.edu.cn (S.W.); 3 Guangxi Academy of Agricultural Sciences, Nanning 530007, China; hqchun1288@163.com; 4 Guangxi Laboratory of Germplasm Innovation and Utilization of Specialty Commercial Crops in North Guangxi, Guangxi Academy of Specialty Crops, Guilin 541004, China; 13707832977@126.com; 5 Tobacco Research Institute, Anhui Academy of Agricultural Sciences (AAAS), Hefei 230001, China; xhahnky2023@163.com; 6 College of Resources and Environment, Zhongkai University of Agriculture and Engineering, Guangzhou 510225, China; riaz1480@hotmail.com

**Keywords:** copper, citrus, chemistry property, microorganism, biomass, nutrients

## Abstract

The prolonged utilization of copper (Cu)-containing fungicides results in Cu accumulation and affects soil ecological health. Thus, a pot experiment was conducted using *Citrus reticulata* cv. Shatangju with five Cu levels (38, 108, 178, 318, and 388 mg kg^−1^) to evaluate the impacts of the soil microbial processes, chemistry properties, and citrus growth. These results revealed that, with the soil Cu levels increased, the soil total Cu (TCu), available Cu (ACu), organic matter (SOM), available potassium (AK), and pH increased while the soil available phosphorus (AP) and alkali-hydrolyzable nitrogen (AN) decreased. Moreover, the soil extracellular enzyme activities related to C and P metabolism decreased while the enzymes related to N metabolism increased, and the expression of soil genes involved in C, N, and P cycling was regulated. Moreover, it was observed that tolerant microorganisms (e.g., *p_Proteobacteria*, *p_Actinobacteria*, *g_Lysobacter*, *g_Sphingobium*, *f_Aspergillaceae*, and *g_Penicillium*) were enriched but sensitive taxa (*p_Myxococcota*) were suppressed in the citrus rhizosphere. The citrus biomass was mainly positively correlated with soil AN and AP; plant N and P were mainly positively correlated with soil AP, AN, and acid phosphatase (ACP); and plant K was mainly negatively related with soil β−glucosidase (βG) and positively related with the soil fungal Shannon index. The dominant bacterial taxa *p_Actinobacteriota* presented positively correlated with the plant biomass and plant N, P, and K and was negatively correlated with plant Cu. The dominant fungal taxa *p_Ascomycota* was positively related to plant Cu but negatively with the plant biomass and plant N, P, and K. Notably, arbuscular mycorrhizal fungi (*p_Glomeromycota*) were positively related with plant P below soil Cu 108 mg kg^−1^, and pathogenic fungi (*p_Mortierellomycota*) was negatively correlated with plant K above soil Cu 178 mg kg^−1^. These findings provided a new perspective on soil microbes and chemistry properties and the healthy development of the citrus industry at increasing soil Cu levels.

## 1. Introduction

Citrus is a type of rutaceae plant with a rich variety of species and is the largest fruit crop in the world [1,2]. Citrus fruit is highly favored by consumers for its unique flavor and a wide range of bioactive substances [3]. However, citrus cultivation regions are primarily located in tropical and subtropical areas characterized by heat and humidity and are also home to many pests and diseases [4]. Chemical control is currently the primary method used to manage diseases and insect pests, with copper (Cu)-containing agents known for their long-lasting and effective germicidal effects, highly appreciated by fruit farmers [5]. In orchard soils, excessive Cu levels have been observed due to pesticide use over the years [6]. Previous studies have shown that soil Cu concentrations in citrus orchards in Southern Florida reached 210.5 mg kg^−1^ and 256.5 mg kg^−1^ in 36 years and 43 years, respectively [7]. The ratios of citrus orchard soil with excessive Cu content in Guangxi, Zhejiang, Hunan, and Jiangxi Provinces, which are the main citrus-producing areas in China, range from 85.3% to 100% [8,9,10]. Additionally, our recent research has also revealed that the proportions of soil sampling points with excessive Cu reached 71.4% in a Guangxi citrus orchard [11]. Therefore, a theoretical framework for the prevention and control of Cu pollution in citrus soil is urgently needed to investigate the effects of the increasing Cu concentrations on soil environmental health.

Cu acts as an essential nutrient element and plays a crucial role in plant growth. However, excessive Cu can inhibit plant physiological and biochemical processes, hinder plant growth, and interfere with fruit development and quality formation [12,13]. Soil fertility refers to the soil capacity to supply plants with the necessary nutrients and biogenic substances. It is a comprehensive measure of the soil’s physical, chemical, and biological properties and serves as a crucial indicator for assessing soil quality [14]. According to previous research, the soil Cu concentration is closely related to soil fertility. For example, as the soil Cu concentration increases, soil organic matter (SOM); available concentrations of nitrogen (N), phosphorus (P), and potassium (K); and the pH value decrease [15]. Cu concentration was negatively correlated with N mineralization when the Cu concentration reached 33.5–107.5 mg kg^−1^ in soil [16]. Soil pH plays a major role in the dissolution and adsorption of Cu compounds, and Cu bioavailability is usually extremely high in strongly acidic soils [17]. Moreover, soil extracellular enzymes are highly responsive to variations in external environmental stress [18], and they can activate various mineral compounds in the soil, thereby enhancing soil effective nutrients and improving soil quality. As soil enzymes are mediated by microbes, the availability of soil nutrients can influence the metabolic activity of microbes, so soil enzyme activity is influenced by soil nutrients [19]. A previous study discovered that there was a significant positive correlation between soil carbon (C), N, and P acquisition enzyme activity and soil soluble organic C and AP, and when SOM accumulation is insufficient, the contents of available nutrients required for microbial metabolic activity decrease, followed by weakening microbial metabolic activity, ultimately reducing enzyme secretion [20]. For example, β-glucosidase is closely linked to the C cycle, and the addition of C can induce microbes to produce more β-glucosidase to acquire the limiting resource [21]. Urease is related to the N cycle, and its activity can indicate the transformation of soil organic N [22]. It has been shown that the activity of soil urease decreases by 77% when the soil Cu^2+^ concentration reaches 250 mg kg^−1^, and a possible explanation is that Cu inhibits soil microorganism growth and reproduction, thereby reducing enzyme synthesis and secretion [23]. Furthermore, the activity of soil alkaline phosphatase and acid phosphatase (ACP) decreased by 48.2% and 15.1% under Cu stress, which may be related to a decrease in microbial biomass [24]. Interesting, vector analysis of the enzyme stoichiometry (vector length and angle) helps to understand the biogeochemical cycling process. The ratio of enzyme activities involved in soil C, N, and P cycling can be transformed into the vector length and angle, which can simultaneously identify the relative nutrient demand of microorganisms without being affected by the total enzyme activity and provide predictions of relative C, N, and P limitations [25,26]. Hence, soil nutrients are able to affect microbial activity, and this, in turn, affects the amount and quality of enzymes secreted by microorganisms.

In addition, microorganisms respond highly to environmental changes in soil and contribute greatly to soil ecological health maintenance and stabilization [27]. Overexposure to Cu can affect the structure, function, and composition of soil microbes, thereby compromising the quality and ecology of soil [28]. It has been shown that low concentrations of heavy metals can promote microbial growth, while high concentrations of heavy metals have an inhibitory effect [27]. High Cu toxicity can negatively impact soil microbial communities by reducing microbial respiration rate and activity, as well as altering the community structure [29]. Furthermore, the continuous buildup of Cu in the soil has been shown to cause a decline both in soil microbial biomass and diversity [30]. Microorganisms play a crucial role in plant growth and nutrient absorption. For instance, certain fungal species of Mortierellomycota are plant pathogens, which can cause necrosis of plant seedlings [29]. Additionally, a close relationship exists between saprophytic fungi and soil C cycling and nutrients decomposition [31]. Hence, soil Cu levels should be examined in relation to soil microbial communities and citrus growth as part of a reciprocal relationship.

In this study, we investigated the impact of soil Cu levels on the characteristics of plant biomass and nutrients concentration, soil nutrients and extracellular enzymes, and soil microbe structure and composition in yellow loam soil planted with Shatangju (*Citrus reticulata* cv. Shatangju). The research utilized high-throughput sequencing techniques for 16S and ITS to analyze these effects. The study aimed to reveal the changes in soil environmental quality associated with the increasing soil Cu levels, providing valuable insights for optimizing Cu nutrient management in orchards, preventing and controlling Cu toxicity in citrus orchards, and providing theoretical references for improving citrus yield and quality.

## 2. Materials and Methods

### 2.1. Experimental Materials and Design

This experiment was conducted from May 2020 to January 2021 in a greenhouse located in the Guilin District of Guangxi Province, China (110°19′58′′ E, 25°16′12′′ N). The origin soil came from the local orchard, and their properties before the experiment were as follows: yellow loam soil with a TCu concentration of 38.00 mg kg^−1^; ACu concentration of 0.83 mg kg^−1^; SOM concentration of 22.59 g kg^−1^; pH of 6.86; and AN, AP, and AK concentrations of 57.75, 32.75, and 354.39 mg kg^−1^, respectively. We selected three-year-old Shatangju (*citrus reticulata* cv. Shatangju) of similar size and consistent growth and added the plants which root systems carried origin soil to the pots with the Cu and then added fertilizer as needed, and the pot size was caliber x barrel height were 54 cm x 46 cm, one pot one plant with 35 kg soil. According to the Cu tolerance of woody plants and the comprehensive range of Cu concentration in citrus orchard soil at home and abroad, the experiment was set up with 5 levels of exogenous Cu with 0, 70, 140, 280, and 350 mg kg^−1^ soil supplied with CuSO_4_·5H_2_O, which were referred to as Cu 38, Cu 108, Cu 178, Cu 318, and Cu 388, respectively. Citrus plants were cultivated in the soil, with each treatment having 3 replicates and each replication with 4 plants. The fertilizers of N, P (P_2_O_5_), and K (K_2_O) in concentrations of 0.10, 0.06, and 0.10 g kg^−1^ (soil) were supplied with CO(NH_2_)_2_, NH_4_H_2_PO_4_, and K_2_SO_4_, and they were applied two times: (1) in May (50% N, 100% P, and 70% K) and (2) in August (50% N and 30% K) [32]. After treatment, the soil was watered every 3 days to maintain a field water capacity of about 70%.

Soil samples were taken from the citrus rhizosphere using a trowel, hammer, and brush, the sampling tools having been disinfected with 75% alcohol at fruit maturity in January 2021. The soil samples in each treatment had 3 replicates, and each replication was a mixed soil coming from 4 plants. Briefly, the large pieces of soil around the root system were removed, and then, the rhizosphere soil on the surface of the root system was brush off by a brush. Subsequently, a portion of the fresh rhizosphere soil was kept in a refrigerator at 4 °C for a short period (within a week) to measure soil extracellular enzyme activity, another part of the fresh soil was stored at −80 °C to determine the soil microorganisms, and the remainder of the fresh soil was air-dried and sieved to determine its chemical properties following Bao [33].

The plant samples contained the tree root, stem, leaf, and fruit. Firstly, the entire tree was pulled out and separated into roots, stems, leaves, and fruits. Plant samples of these 4 parts in each treatment had 3 replicates, and each replicate was a mixed sample coming from 4 plants. Then, the root samples were first cleaned with reverse osmosis (RO) water, followed by soaking in a solution of 5.0 mmol L^−1^ Ca (NO_3_) _2_ for 30 min to resolve Cu adsorbed on the surface of the roots, and finally, the root samples were all cleaned with RO water [34]. The samples of stem, leaf, and fruit were first washed with 0.1% neutral solution for about 30 s, followed by rinsing with clean water, then rinsing with 0.2% HCl solution for about 30 s, and, finally, washed with deionized water for a total cleaning time of less than 2 min. All the cleaning plant samples were placed in an oven and dried to a constant weight. Then, we ground them into powder using a stainless-steel grinder and stored them in a cool and dry place for testing.

### 2.2. Soil Chemical Properties, Enzyme Activities Analysis, and Plant Nutrient Concentrations

The soil pH was measured using a pH electrode (FE20/EL20, Shanghai Mettler Toledo Co., Shanghai, China) in a soil suspension (1:2.5). The potassium dichromate method was used to measure the SOM. Soil AN was determined using the alkaline hydrolysis diffusion method. Soil AP was determined using UV spectrophotometry (TU−1810, Beijing Persee General Instrument Co. Ltd., Beijing, China), and soil AK was assayed using a flame spectrophotometer (AP−1200, Shanghai Precision Instrument Co., Shanghai, China) (Bao, 2000). The soil ACu was extracted with diethylenetriamine pentaacetic acid (DTPA), and the soil TCu was digested by HNO_3_−HCl−HF (4:1:1). The Cu concentration was then measured using an atomic absorption spectrometer (Z-2000, HITACHI, Tokyo, Japan) [35].

The soil extracellular enzyme activities, including α-glucosidase (αG), β−glucosidase (βG), β−cellobiohydrolase (CBH), β−xylosidase (βX), N−acetyl−glucosaminidase (NAG), and acid phosphatase, were determined using fluorescence microplate enzyme detection technology. As a substrate, 4−methylumbelliferyl (4−MUB) was used to label the hydrolase activity, and wavelengths of 365 nm and 450 nm (Scientific Fluoroskan Ascent FL, Thermo, Potsdam, Germany) were used for detection [36]. Fe^3+^ chelate reductase (FRO) was determined using enzyme-linked immunosorbent assay (ELISA) kits (Meimian Biotechnology Co. Ltd., Shanghai, China). The soil extracellular enzyme vector measured as follows: vector length = SQRT (X^2^ + Y^2^), vector angle = DEGREES (ATAN2 (X, Y)), X = C/(C + P); Y = C/(C + N); C/(C + P): (βG + CBH + βX + αG)/(βG + CBH + βX + αG + ACP); C/(C + N): (βG + CBH + βX + αG)/(βG + CBH + βX + αG + NAG).

The plant Cu concentration was measured by mixed acid (HNO_3_:HClO_4_ = 4:1, *v*/*v*) digestion and analyzed using an atomic absorption spectrometer (Z−2000, HITACHI, Tokyo, Japan) [35]. Plant N, P, and K were measured by H_2_SO_4_–H_2_O_2_ digestion and analyzed through distillation, molybdenum-antimony colorimetry, and flame photometry, respectively [14].

### 2.3. DNA Extraction, PCR Amplification, and High-Throughput Sequencing

We used a FastDNA^®^ Spin Kit for Soil (MP Biomedicals, Norcross, GA, USA) to extract the soil total DNA. The purity and concentration of the DNA were examined by a NanoDrop 2000 (Thermo Fisher Scientific, Waltham, MA, USA), and the quality of DNA was detected with 1% agarose gels electrophoresis (DYY-6C, Beijing Lliuyi Biotechnology Co., Ltd., Beijing, China). The amplification of the V3–V4 region of bacteria was performed with primers 338F (5′-ACTCCTACGGGAGGCAGCAG-3′) and 806R (5′−GGACTACHVGGGTWTCTAAT−3′) [37], and the amplification of the hypervariable ITS region of fungi was conducted with primers ITS1F (5′−CTTGGTCATTTAGAGGAAGTAA−3′) and ITS2R (5′−GCTGCGTTCTTCATCGATGC−3′) [38]. PCR products were recovered by 2% agarose gels, purified with Axy Prep DNA Gel Extraction Kits (Axygen Biosciences, and Union City, CA, USA), and quantified using a Quantus™ Fluorometer (Promega, Solana Beach, CA, USA). The PCR amplification and library construction were done as described by Zhang et al. [39]. We sequenced 2 × 300 bp paired ends on an Illumina MiSeq (Illumina, San Diego, CA, USA) with the assistance of Shanghai Majorbio Bio-pharm Technology Co., Ltd., Shanghai, China.

### 2.4. Data Analysis

Quality control and splice of the raw sequences were conducted by fastp software (https://github.com/OpenGene/fastp/ (accessed on 4 July 2024), version 0.20.0) [40] and FLASH software (http://www.cbcb.umd.edu/software/flash/ (accessed on 4 July 2024), version 1.2.7) [41]. In order to eliminate the effect of the sequencing depth on the subsequent analysis, all samples were resampled randomly based on 48,587 reads by using the minimum number of sequences. By using UPARSE (http://drive5.com/uparse/ (accessed on 4 July 2024), version 7.1) [42], the OTUs (operational taxonomic units) were clustered based on 97% similarity. Lastly, all sequences were annotated using the RDP classifier (http://rdp.cme.msu.edu/ (accessed on 4 July 2024), version 2.11), with a 70% confidence threshold, and the bacteria and fungi were aligned with the Silva 128 database and Unite 7.0 database, respectively [43].

We used Mothur software (http://www.mothur.org/wiki/Calculators/ (accessed on 4 July 2024), version 1.30.1) to evaluate the alpha diversity index of Shannon with Student’s *t*-tests based on the OTUs [44]. R software (version 3.3.1) with the package “vegan” was used to operate the principal coordinate analysis (PCoA), package “lavaan” was used to conduct the redundancy analysis (RDA), and package “pheatmap” was used to perform the heatmap. In order to identify the different biomarkers of citrus rhizospheres under different soil Cu levels, taxa with differential abundances were evaluated using a factorial Kruskal–Wallis sum rank test (*p* < 0.05) and Linear discriminant analysis Effect Size (LEfSe, LDA score > 3.5). Biomarkers’ importance was shown in the cladograms by depicting the identified taxa. LEfSe analyses were conducted online in the ‘LEfSe’ (http://huttenhower.sph.harvard.edu/galaxy/root?tool_id=lefse_upload/ (accessed on 4 July 2024). To further explore the functional classification of the rhizosphere microbial communities, PICRUSt2 (https://github.com/picrust/picrust2/ (accessed on 4 July 2024), version 2.2.0) and FUNGuild (http://www.funguild.org/ (accessed on 4 July 2024), version 1) were operated to analyze bacteria and fungi group functions, and then, the C, N, and P metabolism functional genes in rhizosphere bacteria were analyzed based on KO values by the KEGG database; this analysis was performed on the Tutools platform (https://www.cloudtutu.com/ (accessed on 4 July 2024)), a free online data analysis website. Procrustes analysis was used to explore the relationship between microbial communities (bacteria and fungi) and environmental factors. The analyses were carried out with the ‘vegan’ package and visualized with ‘ggplot2’ in R. Additionally, co-occurrence network analysis of the microbial community (bacteria and fungi) and environmental factors in low (Cu 108 mg kg^−1^) and high (Cu 178, 318, and 388 mg kg^−1^) Cu levels were performed with abundant OTUs (top 200) and indicators of plant biomass, plant nutrient concentrations, and chemical properties and soil enzyme activities.

The soil chemical properties and soil enzyme activities and plant nutrient concentrations between different treatments in soil were examined using one-way analysis of variance using Duncan’s test (*p* < 0.05) by SPSS (IBM Corp, Armonk, NY, USA). We reported all results as the mean + standard deviation (*n* = 3). Different lowercase letters (a, b, c, d, etc.) among different treatments indicated significant differences (*p* < 0.05).

## 3. Results

### 3.1. Soil Chemical Properties and Extracellular Enzymes

The soil TCu and ACu concentrations increased significantly (*p* < 0.05) as the soil Cu levels increased (Table 1). Compared to the Cu 38 treatment, the soil TCu and ACu concentrations were elevated by 5.07 and 245.06 times, respectively, the Cu 388 treatment. Additionally, the soil chemical properties exhibited variations under different soil Cu levels (Table 1). Compared to the Cu 38 treatment, the SOM and AK concentrations significantly (*p* < 0.05) increased by 6.79–22.12% and 8.05–14.78%, respectively (Table 1). However, with the increase in soil Cu levels, the soil pH decreased by 0.10–0.17 units, while the soil AN and AP concentrations were notably reduced by 15.55% and 63.06% in the Cu 318 treatment and by 29.38% and 69.35% in the Cu 388 treatment (Table 1). These results suggested that, as the soil Cu levels increase, the soil TCu, ACu, SOM, AK concentrations, and soil pH increased, while the soil AP and AN concentration decreased.

Different soil Cu level treatments had varying effects on the FRO, αG, βG, CBH, βX, NAG, and ACP (Table 2). In more detail, compared to the Cu 38 treatment, the activity of FRO increased by 1.62 to 2.36 times (Table 2). The αG, βG, CBH, and βX in the Cu 388 treatment were notably reduced by 82.76%, 23.85%, 15.76%, and 16.50%, respectively, compared to the Cu 38 treatment (Table 2). With the increasing soil Cu levels, the activity of NAG first decreased and then increased, with the highest activity of NAG observed in the Cu 178 treatment (Table 2). Regarding the soil P-related enzyme ACP, compared to the Cu 38 treatment, the activity of ACP significantly (*p* < 0.05) decreased by 51.47% and 42.16% in the Cu 318 and Cu 388 treatments, respectively (Table 2). Moreover, in the results of the enzyme stoichiometry, the vector length was significantly (*p* < 0.05) reduced by 7.58% to 15.80% with the increasing soil Cu levels (Figure S1A). Similar to the vector length results, the vector angles were all higher than 45° and significantly (*p* < 0.05) reduced by 2.88% to 13.30% in the higher soil Cu levels compared to the Cu 38 treatment (Figure S1B). These results suggested that, with the increasing soil Cu levels, the soil extracellular enzymes activities related to C and P metabolism decreased, but those related to N and Fe metabolism increased.

### 3.2. Plant Growth and Nutrients Uptake

**Figure 1:**
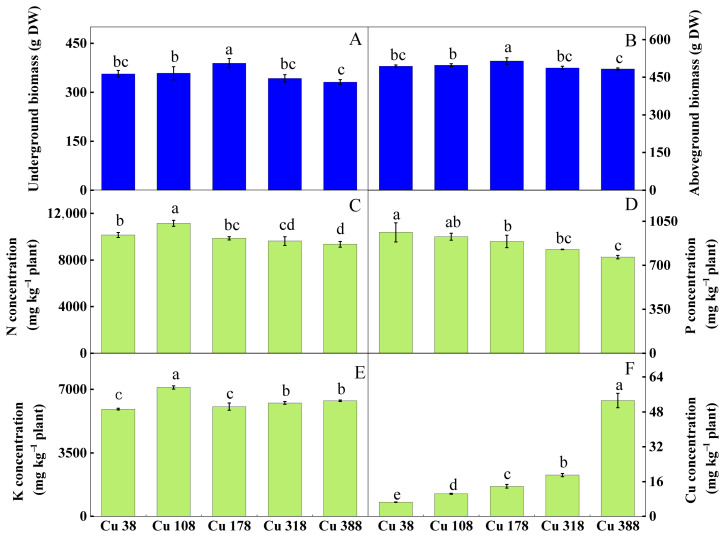
Plant biomass (**A**,**B**) and nutrient concentrations (**C**–**F**) of *Citrus reticulata* cv. Shatangju under different soil Cu levels. Note: Different lowercase letters indicate significant differences among the treatments by Duncan-test (*p* < 0.05, *n* = 3).

The citrus dry biomass underground and aboveground in the Cu 108 treatment was higher than that in the Cu 38 treatment, while a decreasing trend was observed in the Cu 388 treatment compared to the Cu 38 treatment (Figure 1A,B). Moreover, the nutrient concentrations in the plants varied in different soil Cu levels. The plant N concentrations were higher in the Cu 108 treatment but were lower in the Cu 318 and Cu 388 treatments, and the plant P concentrations exhibited a declining pattern as the soil Cu levels increased. However, the plant Cu concentrations increased with the rising soil Cu levels. Additionally, an increase in soil Cu also increased the plant K concentrations.

### 3.3. Soil Microbial Diversity, Composition, and Markers

**Figure 2:**
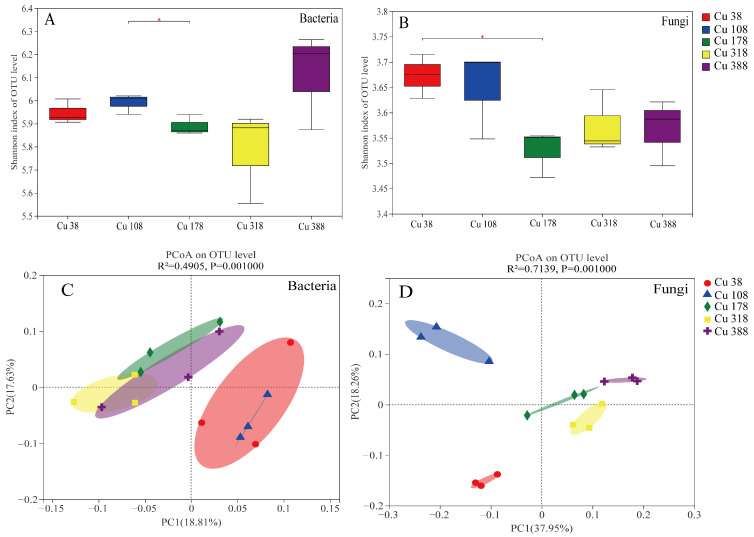
Diversity in soil with five Cu treatments of bacterial and fungal microbial communities in *Citrus reticulata* cv. Shatangju rhizosphere. Note: (**A**,**B**) indicated a Shannon index analysis of the rhizosphere bacteria and fungi, and (**C**,**D**) indicated a PCoA diversity analysis of the bacteria and fungi. Asterisks denoted significant differences (* *p* < 0.05).

The number of OTUs for the bacteria and fungi reads reached approximately 30,000 and 45,000, respectively, and both approached saturation (Figure S2). This suggested that the sequencing reads were sufficient and reliable. The results of the alpha diversity are presented in Figure 2A,B, revealing a significant difference in the Shannon index of the fungi between Cu 38 and Cu 178. Additionally, we examined the differences in microbial composition among different soil Cu levels using PCoA. Based on the Bray–Curtis distance, we found that the microbial community of the low Cu treatment (Cu 108) was similar to the Cu 38 treatment, while the other three higher Cu treatments (Cu 178, Cu 318, and Cu 388) formed a cluster distinct from the Cu 38 treatment (Figure 2C,D). Furthermore, the degree of separation observed in the fungal communities was greater than that observed in the bacteria. Based on the PCoA results, we defined the Cu 38 treatment as the control check treatment (short for CK); the Cu 108 treatment as the low Cu treatment (short for Low Cu); and the group of Cu 178, Cu 318, and Cu 388 as the high Cu treatment (short for High Cu).

**Figure 3:**
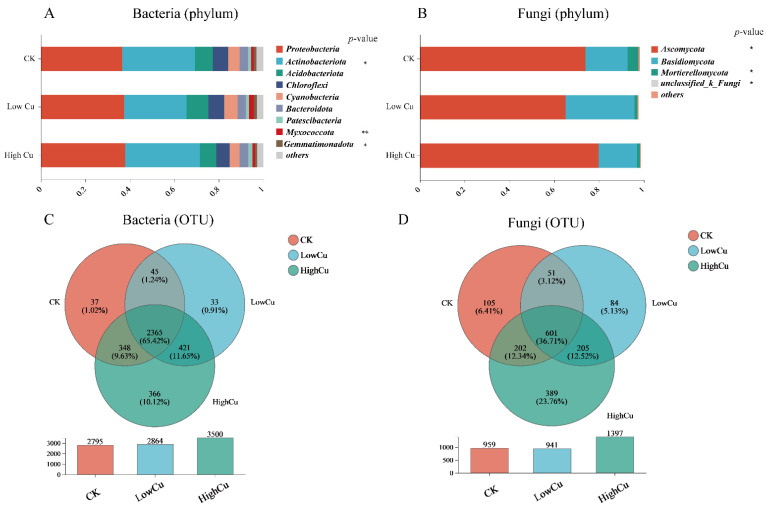
Bacterial and fungal community composition ((**A**,**B**): Phylum level; (**C**,**D**): OTUs level) in *Citrus reticulata* cv. Shatangju rhizosphere. Note: CK indicated soil with 38 mg kg^−1^ Cu; Low Cu indicated soil with 108 mg kg^−1^ Cu; and High Cu indicated soil with 178 mg kg^−1^, 318 mg kg^−1^, and 388 mg kg^−1^ Cu, respectively. Asterisks denoted significant differences (* *p* < 0.05, and ** *p* < 0.01).

At the phylum level, *p_Proteobacteria*, *p_Actinobacteriota*, *p_Acidobacteriota*, and *p_Chloroflexi* were the main bacterial taxa, accounting for approximately over 80% of the bacterial community (Figure 3A). Furthermore, significant differences were observed in *p_Actinobacteriota*, *p_Myxococcota*, and *p_Gemmatimonadota* at different soil Cu levels. In addition, *p_Ascomycota*, *p_Basidiomycota*, and *p_Mortierellomycota* were the main fungal taxa at the phylum level, comprising 95% of the fungal community (Figure 3B). There were also significant differences in *p_Ascomycota* and *p_Mortierellomycota* at different soil Cu levels. As shown in the Venn diagram for bacteria, there were 2365 shared OTUs (65.42%), and 37 (1.02%), 33 (0.91%), and 366 (10.12%) OTUs were specifically found in the CK, low Cu, and high Cu treatments, respectively (Figure 3C). Additionally, the Venn diagram for fungi showed 601 shared OTUs (36.71%) and 105 (6.41%), 84 (5.13%), and 389 (23.76%) OTUs specifically found in the CK, low Cu, and high Cu treatments, respectively (Figure 3D). These results suggested that the high Cu treatment had a larger number of new microbial communities, and the rate of special OTUs for the fungi was much higher than that for the bacteria. This might explain the notable disparity (β diversity) observed in the soil microbes in the high Cu treatment due to the fungi exhibiting a particularly pronounced susceptibility to soil Cu treatment (Figure 3C,D).

**Figure 4:**
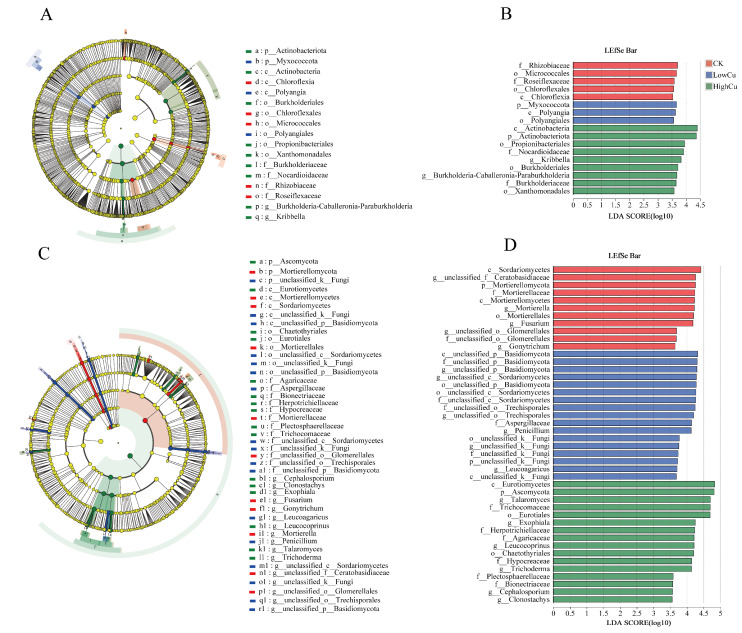
Cladogram showing the phylogenetic distribution of the bacterial (**A**) and fungal (**C**) lineages, and bar charts showing the biomarkers of the bacterial (**B**) and fungal (**D**) taxon. Note: Red, blue, and green dots represented bacteria and fungi with significantly enriched abundances. CK indicated soil with 38 mg kg^−1^ Cu; Low Cu indicated soil with 108 mg kg^−1^ Cu; and High Cu indicated soil with 178 mg kg^−1^, 318 mg kg^−1^, and 388 mg kg^−1^ Cu, respectively.

According to the LEfSe analysis of bacteria, the cladogram shown that one class, two orders, and two families were enriched in the CK treatment, while one phylum (*Myxococcota*), one class, and one order were enriched in the low Cu treatment. The high Cu treatment exhibited enrichment in one phylum (*Actinobacteria*), one class, three orders, two families, and two genera (Figure 4A). Moreover, as shown in the LDA score, the biomarkers of the bacteria communities at different soil Cu levels were identified (Figure 4B). The top five biomarkers of bacteria enriched in the CK treatment were *f_Rhizobiaeae*, *o_Micrococcales*, *f_Roseiflexaceae*, *o_Chloroflexales*, and *c_Chloroflexia*. In the low Cu treatment, the top three biomarkers of bacteria were *p_Myxococcota*, *c_Polyangia*, and *o_Polyangiales*. For the high Cu treatment, the top five biomarkers of bacteria were *c_Actinobacteria*, *p_Actinobacteriota*, *o_Propionibacteriales*, *f_Nocardiodioidaceae*, and *g_Kribbella*.

In addition, the LEfSe analysis of the fungi revealed that, in the cladogram, one phylum (*Mortierellomycota*), two classes, one order, two families, and five genera were concentrated in the CK treatment. The low Cu treatment displayed enrichment in one phylum (unclassified), two classes, three orders, five families, and six genera. Meanwhile, the high Cu treatment exhibited enrichment in one phylum (*Ascomycota*), one class, two orders, six families, and six genera (Figure 4C). Based on the LDA score, the biomarkers of the fungal taxa at different soil Cu levels were identified (Figure 4D). The top five biomarkers of the fungi concentrated in the CK treatment were *c_Sordariomycetes*, *g_unclassified_f_Ceratobasidiaceae*, *p_Mortierellomycota*, *f_Mortierellaceae*, and *c_Mortierelloycetes*. In the low Cu treatment, the top five biomarkers of the fungi were *c_unclassified_p_Basidiomycota*, *f_unclassified_p_Basidiomycota*, *g_unclassified_p_Basidiomycota*, *g_unclassified__c_Sordariomycetes*, and *o_unclassified_p_Basidiomycota*. In the high Cu treatment, the top five biomarkers of the fungi were *c_Eurotiomycetes*, *p_Ascomycota*, *g_Talaromyces*, *f_Trichocomaceae*, and *o_Eurotiales*. Furthermore, the phylum proportions at various soil Cu levels were significantly different (Figure S3).

### 3.4. Function Classification of Rhizosphere Microbial Communities

**Figure 5:**
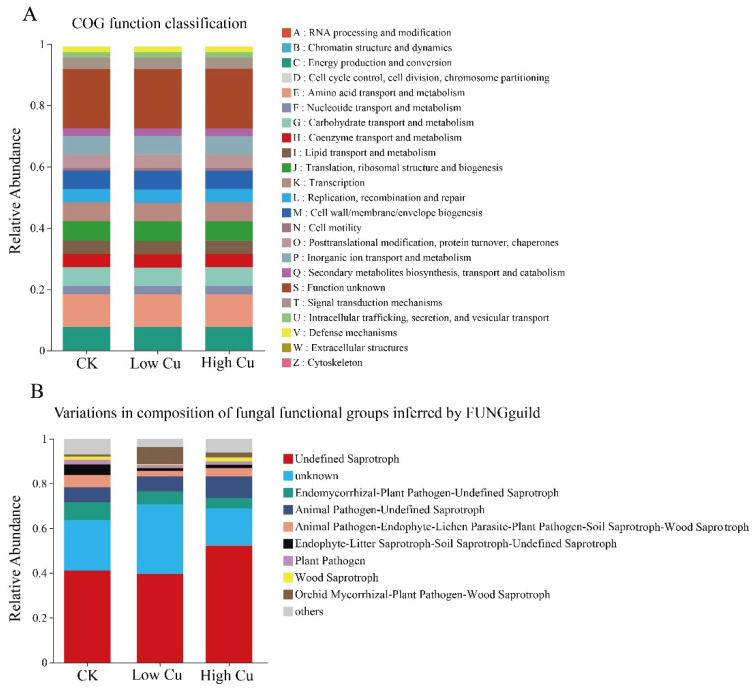
Functional classification of bacteria (**A**) and fungi (**B**) in *Citrus reticulata* cv. Shatangju rhizosphere. Note: CK indicated soil with 38 mg kg^−1^ Cu; Low Cu indicated soil with 108 mg kg^−1^ Cu; and High Cu indicated soil with 178 mg kg^−1^, 318 mg kg^−1^, and 388 mg kg^−1^ Cu, respectively.

The functions classification of the bacteria communities mainly included amino acid transport and metabolism, energy production and conversion, carbohydrate transport, and metabolism, translation, ribosomal structure and biogenesis, transcription, cell wall/membrane/envelope biogenesis, inorganic ion transport and metabolism, and more. Generally, the function classification of the bacterial communities showed no significant difference between different treatments (Figure 5A). However, based on the analysis of functional genes related to C, N, and P metabolism, significant differences in gene expression were observed at different soil Cu levels (Figure S4). Most genes of microorganisms involved in C and N metabolism displayed downregulation in the low Cu treatment and upregulation in the high Cu treatment. Interestingly, with the increasing soil Cu levels, C degradation genes *cdhD* and *cdhE* were downregulated, while C fixation genes *aclA* and *aclB* were upregulated. The methane production gene *mxaF* showed upregulation, while methane oxidation genes *pmoA*, *pmoB*, and *pmoC* were downregulated (Figure S4A). Moreover, with the increasing soil Cu levels, nitrification genes *amoA*, *amoB*, and *amoC* were downregulated, while denitrification genes *norB* showed upregulation and *narG* showed downregulation. Genes *nirB*, *nirD*, and *nirK* were downregulated in the low Cu treatment but upregulated in the high Cu treatment. N fixation genes *nifD* and *nifH* were downregulated, and dissimilatory N reduction genes *napA* and *napB* were upregulated (Figure S4B). In addition, a large number of genes (*phnE*, *phnD*, *phnC*, *phnM*, *phnL*, *phnK*, *phnJ*, *phnI*, *phnG*, *phnH*, *phoR*, and *phoU*) associated with organic P mineralization were upregulated in the low Cu treatment but downregulated in the high Cu treatment. Genes *ppk1* associated with inorganic P biosynthesis and *ppx* associated with inorganic P hydrolysis were downregulated in the low Cu treatment but upregulated in the high Cu treatment. The gene *gcd* involved in inorganic P solubilization was upregulated with the increasing soil Cu levels (Figure S4B).

According to the fungal functional groups (Figure 5B), as the soil Cu levels increased, the functional abundance of animal pathogens, endophytes, lichen parasites, plant pathogens, soil saprotrophs, and wood saprotrophs decreased significantly. In contrast, the functional abundance of orchid mycorrhizal and wood saprotroph fungi increased markedly in the low Cu treatment. However, the functional abundance of animal pathogens and undefined saprotrophs significantly increased in the high Cu treatment.

### 3.5. Co-Occurrence Associations between Microorganism and Plant Biomass and Plant Nutrients Concentration

**Figure 6:**
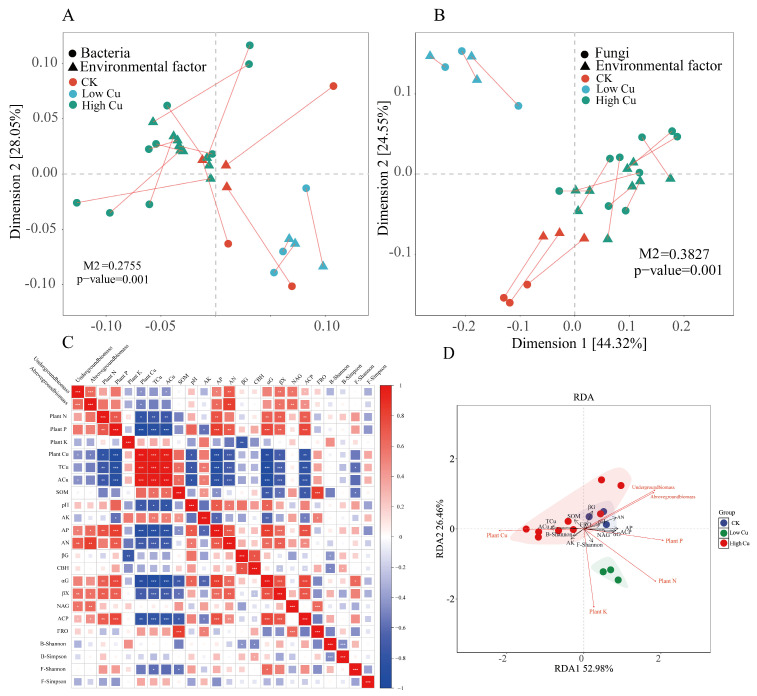
The effects of environmental factors and microorganisms on plant biomass and nutrient concentrations. Note: (**A**) Procrustes analysis of bacteria (*Shannon index*), and environmental factors with plant biomass and nutrient concentrations. (**B**) Procrustes analysis of fungi (*Shannon index*), and environmental factors with plant biomass and nutrient concentrations. (**C**) The correlation between environmental factors and citrus biomass and nutrient concentration indicators. (**D**) Redundancy analysis of the citrus biomass and nutrient concentration and environmental factors. CK indicated soil with 38 mg kg^−1^ Cu; Low Cu indicated soil with 108 mg kg^−1^ Cu; and High Cu indicated soil with 178 mg kg^−1^, 318 mg kg^−1^, and 388 mg kg^−1^ Cu, respectively. Asterisks denoted significant differences (* *p* < 0.05, ** *p* < 0.01, *** *p* < 0.001).

The Procrustes analysis, redundancy analysis, and co-occurrence network analysis were employed to establish a connection between microorganisms and plant biomass and plant nutrient concentrations. The Procrustes analysis provided an overview of the soil chemistry and microbe communities in relation to plant biomass and nutrient concentrations. The values of M2 (bacteria: 0.2755, fungi: 0.3827) and *p* (bacteria: 0.001, fungi: 0.001) are displayed in Figure 6A,B, which showed that the plant biomass and nutrient concentrations were closely related to the soil chemistry and microbe communities. The correlation heatmap displayed that the underground and aboveground biomass were significantly negative with soil ACu but positive with soil AP, AN, βX, NAG, and ACP. Plant N and plant P were significantly negative with soil TCu and ACu but positive with soil AP, AN, αG, βX, and ACP. Plant K was only significantly negative with soil βG. Moreover, plant Cu was significantly positive with soil TCu and ACu but negative with soil pH, AP, AN, αG, βX, and ACP (Figure 6C). The redundancy analysis showed that the underground and aboveground biomass were mainly positively correlated with soil AN and AP, and plant N and plant P were mainly positively correlated with soil AP, AN, and ACP. Plant K was mainly negative with the soil βG and positive with the soil fungal Shannon index (Figure 6D). The results showed that the biomass and nutrient concentrations of the plants were affected differently by the soil chemical properties and microorganisms.

**Figure 7:**
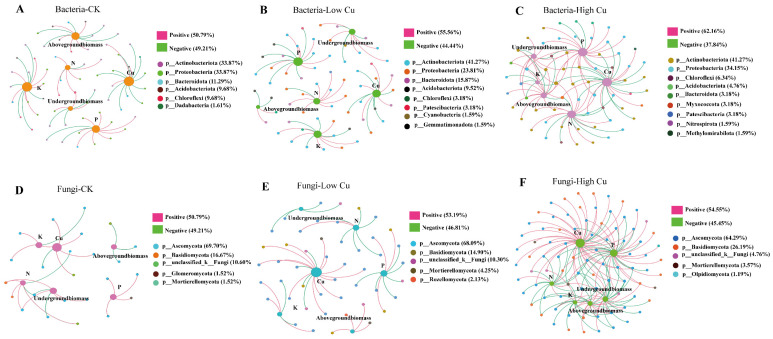
Correlation network diagram of the soil microorganisms and plant biomass and nutrient concentrations. Note: The size of the graph nodes represented the abundance of the species, and different colors represented different species. The color of the connecting line represented positive and negative correlations: the red line indicated a significant positive correlation, and the green line indicated a significant negative correlation. The line thickness indicated the size of the correlation coefficient, Pearson’s correlation analysis was used, and the correlation coefficient in the graph defaulted to a value of *p* < 0.05. The number of lines indicated the degree of closeness between nodes. CK indicated soil with 38 mg kg^−1^ Cu; Low Cu indicated soil with 108 mg kg^−1^ Cu; and High Cu indicated soil with 178 mg kg^−1^, 318 mg kg^−1^, and 388 mg kg^−1^ Cu, respectively.

To investigate the connection between soil microorganisms and plant biomass and plant nutrient concentrations in more detail, a co-occurrence network was performed using the top 200 OTUs based on relative abundance (Figure 7). For the bacteria, *Actinobacteriota* had the most significant impact on the plant biomass and nutrient concentrations. In the CK treatment, the bacteria had a close relationship with the plant Cu and K concentrations. It is worth noting that *Actinobacteriota* was mainly negatively correlated with the plant biomass and K and P concentrations, while *Proteobacteria* was mainly negatively correlated with the plant Cu concentration but positively correlated with the plant K and P concentrations (Figure 7A). In the low Cu treatment, the bacteria had a close relationship with the plant Cu, P, and K concentrations. In detail, *Actinobacteriota* was mainly negatively correlated with the plant Cu concentration but positively correlated with the plant N, P, K, and biomass, while *Proteobacteria* was mainly positively related to the plant N and K concentrations but negatively related to the plant Cu concentration (Figure 7B). In the high Cu treatment, the bacteria had a close relationship with the Cu, P, and N concentrations in the plants. In detail, *Actinobacteriota* was primarily negatively related to the plant Cu concentration but positively related to the plant N and P concentrations and biomass, while *Proteobacteria* was mainly negatively correlated with the plant Cu concentration but positively correlated with the plant P concentration and biomass (Figure 7C). These results indicated that the dominant bacterial taxa *Actinobacteriota* was mainly positively correlated with the plant biomass and N, P, and K concentrations but mainly negatively correlated with the plant Cu concentration with the increasing soil Cu levels.

Regarding the fungi, in terms of impact on plant growth and nutrient uptake, *Ascomycota* had the most significant influence. In the CK treatment, the fungi had a close relationship with the plant Cu concentration. *Ascomycota* was positively associated with the plant Cu, P, and K concentrations and underground biomass (Figure 7D). In the low Cu treatment, the fungi had a close relationship with the plant Cu concentration. *Ascomycota* was mainly positively associated with the plant Cu and K concentrations but negatively related to the plant biomass and N and P concentrations (Figure 7E). In the high Cu treatment, the fungi had a close relationship with the plant Cu and P concentrations. *Ascomycota* was primarily positively associated with the plant Cu concentration but negatively related to the plant biomass and N, P, and K concentrations. *Basidiomycota* was mainly positively correlated with the plant Cu and P concentrations (Figure 7F). These results suggest that the dominant fungal taxa *Ascomycota* was mainly negatively correlated with the plant biomass and N, P, and K concentrations but positively correlated with the plant Cu uptake with the rise in the soil Cu levels.

## 4. Discussion

### 4.1. Soil Chemistry Properties under Different Soil Cu Levels

An increase in the soil Cu levels resulted in a higher accumulation of SOM and soil AK but led to a decrease in soil AN and AP and a reduction in soil pH (Table 1). As the soil Cu levels increased, soil elements cycling also had significant changes (Figure S4). Soil enzyme activities have alterations under the influence of heavy metal stress, subsequently impacting soil nutrients cycling [45]. The increase in the SOM concentration might be attributed to a decrease in soil microbial activities caused by high Cu levels (Cu 178, Cu 318, and Cu 388), leading to a decrease in microbial carbon demand and ultimately delaying the mineralization rate of the SOM [46]. It can be inferred that the decrease in the length of the soil extracellular enzyme vector is a result of a high Cu concentration, which inhibited the uptake of C nutrients by soil microbes (Figure S1). Moreover, a decline in saprotrophic abundance could also be a contributory factor (Figure 5B), as they play a vital role in nutrients cycling between soil and litter, closely related to C cycling and nutrients decomposition [31]. In addition, the upregulation of C fixation genes (*aclA* and *aclB*) and the downregulation of C degradation genes (*cdhD* and *cdhE*) with the increase in the soil Cu levels (Figure S4A) also supported C cycling hindrance and led to the SOM increase. Furthermore, the soil AP concentration decline could be attributed to the rapid depletion of AP by soil microorganisms (Figure S1). The activity of ACP decreased with the increasing soil Cu levels, possibly due to Cu^2+^ binding to phosphatase, resulting in enzyme protein degradation and a subsequent decrease in activity [47]. This decrease may also be attributed to the reduction in soil AP concentration and the availability of the phosphatase substrate [48]. Additionally, many genes associated with organic P mineralization were upregulated in the low Cu treatment but downregulated in the high Cu treatments (Figure S4B). These findings further support that the decrease in soil AP concentration is related more to organic P mineralization. The decrease in soil AN concentration with the increasing soil Cu levels might be associated with the rise in NAG enzyme activity (Table 2). NAG enzymes promote the mineralization of organic matter and release N for plant and microbial utilization, ultimately leading to a reduction in the soil AN concentration [49]. Furthermore, the upregulation of denitrification genes (*norB*) and dissimilatory N reduction genes (*napA* and *napB*), along with the downregulation of N fixation genes (*nifD* and *nifH*) with the increasing soil Cu levels (Figure S4B), also supported the idea that the decrease in soil AN concentration might be associated with N decomposition increase and synthesis reduction. The decrease in soil pH may be a stress response of plant roots to Cu stress. Root exudates, such as amino acids, organic acids, clays, and surfactants, can interact with heavy metals and mitigate their toxicity [50,51]. This study also discovered that the activity of FRO increased with the increasing soil Cu levels (Table 1). One possible explanation might be related to FRO converting soil Cu^2+^ into Cu^+^, thereby enhancing Cu bioavailability [52]. Therefore, under different soil Cu levels, soil microbial activities affected nutrients cycling and metabolism, subsequently influencing the soil nutrient status.

### 4.2. Different Cu-Tolerant Biomarkers and Functional Groups Identified under Different Soil Cu Levels

The structure of the soil microbial community is widely recognized as a highly sensitive biological indicator for assessing the stability of soil ecosystems and predicting changes in the environmental quality [53,54]. In this study, the compositions of the fungal communities and the response of different fungal species to the Cu levels exhibited greater variations compared to the bacteria, as depicted in Figure 2. Additionally, as the soil Cu levels increased, the OTU number also increased, and the alterations in the fungi became more pronounced (Figure 3). Furthermore, under different soil Cu levels, the fungal shared OTU ratio was only 36.71%, which was much lower than that of the bacteria (65.42%). However, the fungal-specific OTU ratios were much higher than those of the bacteria (Figure 3). Meanwhile, the LAD analysis displayed that the significant difference in OTU numbers in the fungi was also obviously greater than that in the bacteria (Figure 4). This evidence indicated that the fungi were more sensitive to Cu toxicity. This response may be attributed to varying sensitivities of different soil microorganisms (fungi and bacteria) towards Cu^2+^ concentrations [55]. When exposed to heavy metal stress, fungi experience an increase in reactive oxygen species and proteases, leading to an acceleration in protein breakdown and degradation [56]. Nevertheless, bacteria might have a greater ability to adjust to metal stressors compared to fungi, potentially because of their quicker metabolism and more extensive resource utilization [57].

Generally, plant growth-promoting bacteria were enriched in the rhizosphere of low heavy metal accumulators, whereas bacteria-enhancing plant tolerance flourished in the rhizosphere of high heavy metal accumulators [58]. Following the application of heavy metals to the soil, it can be observed that sensitive microorganisms perish while tolerant microorganisms persist due to their ability to produce tolerance mechanisms [48]. At present, it is less known how different soil Cu levels influence citrus plants’ rhizosphere bacteria. It has been shown that *Proteobacteria*, *Chloroflexi*, *Gemmatimonadetes*, *Planctomycetes*, and *Nitrospirae* are Cu-tolerant bacteria in Cu-polluted soil [59]. Previous studies have found that *Proteobacteria* and *Actinobacteria* possess a high capacity for metal tolerance [60]. Specifically, in heavy metal-contaminated areas, *Proteobacteria* have proven to be the most metal-tolerant bacteria [61]. It has been found that *Microbacterium* (*Actinobacteria*) had an impact on the absorption of heavy metals in the rhizosphere environment, which can be attributed to the production of extracellular polymers by *Microbacterium* [62]. In this study, *Proteobacteria*, *Actinobacteriota*, *Acidobacteriota*, *Cyanobacteria*, *Chloroflexi*, and *Gemmatimonadota* were the main species that increased with the rising soil Cu levels (Figure 3), indicating that most of them possessed a strong Cu resistance ability. However, *Myxococcota* is not quite resistant to heavy metal toxicity [63], resulting in a decrease in its abundance during the high Cu treatment (Figure 3A). Additionally, there were several bacteria, such as *Lysobacter* and *Sphingobium*, enriched in the high Cu treatment soil (Figure S3). Since *Sphingobium* can improve glutathione biosynthesis and oxidative stress resistance, plants can better tolerate metals [64]. Moreover, *Sphingobium* might be a potential host for Cu resistance genes *copA* and *pcoA* [65], and *Lysobacter* might be a potential host for Cu resistance genes *copB* and *czcA* [66].

The soil Cu level increases significantly affected the composition of the fungal community (Figure 3B). Fungi possess diverse physiological characteristics that contribute to their varying response patterns. In this study, the composition of the fungal community exhibited significant variations under different soil Cu levels compared to the CK treatment. These observed variations might be attributed to the selection of tolerant species, while sensitive species were suppressed [67]. *Ascomycota*, the dominant fungi displaying strong Cu resistance under high Cu treatment (Figure 3B), can be attributed to the presence of an extracellular polysaccharide layer outside the cell wall, which isolates metals from the external environment [68]. LEfSe analysis is a reliable approach for identifying biomarkers within microbial communities [69]. Some biomarkers can indirectly reduce the toxicity of heavy metals at high Cu levels through processes such as precipitation, chelation, or degradation [40]. The findings indicated that various organisms served as indicators of Cu tolerance at varying levels of Cu contamination (Figure 4). For instance, in this study, *Aspergillaceae* was identified as a potential biomarker in the low Cu (Cu 108) treatment with remarkable Cu resistance. *Aspergillus flavus* (*Aspergillaceae*) isolated from dye industrial sludge exhibited a greater resistance to Cu compared to other isolates [70]. *Aspergillus* tolerated Cu by upregulating the expression of the P-type ATPase *CrpA* through its transcription factor *AceA* to detoxify Cu [71]. Moreover, *Penicillium* was also considered a biomarker (Figure 4). Several studies have shown that *Penicillium* can effectively remove Cu as a biosorbent [72] and is a keystone taxon in Cu-polluted environments, with the potential to remove heavy metals, detoxify, and stimulate the resistance of fungal communities to Cu pollution [73]. Fungal communities with a high Cu tolerance will contribute to the stability of organism communities [74]. These findings suggested that these biomarkers had a significant impact on Cu-polluted soils and can potentially thrive in environments with varying soil Cu levels.

The abundance of mycorrhizal fungi increased at the low Cu treatment (Figure 5B). Fungi play a vital role in mineral nutrients absorption, reducing metal element toxicity and ultimately promoting plant growth [75]. Thus, the increase in nutrient concentrations and plant biomass in the low Cu treatment (Figure 1) may be highly related to the increase in mycorrhizal fungi abundance. Therefore, in the low Cu treatment, mycorrhizal fungi appear to be more effective in enhancing plant resistance to Cu. In addition, the increase in fungal relative abundance (OTUs) in the high Cu treatment potentially indicated a decline in the overall soil health status (Figure 3D), because the abundance and diversity of fungi exhibit a negative correlation with the overall health status of the soil [15]. For example, the relative abundance of animal pathogens increased at the high Cu levels (Figure 5B). Previous studies have found that specific fungal species in the *Mortierella* (*Mortierellomycota*) class can act as plant pathogens and lead to necrosis in plant seedlings [29]. Within the fungal community thriving in soil, certain fungi possess the ability to directly induce cell death in plants or produce metabolic toxins, thereby exerting a direct impact on the overall health of the plant [76,77]. Previous studies have demonstrated that a decline in soil quality can lead to the emergence of pathogenic fungi in the soil, resulting in detrimental effects such as root epidermal cell necrosis, root tip decay, inhibited growth of lateral roots, and reduced functionality of root hairs [76,78]. In summary, the fungi were more susceptible to Cu toxicity than the bacteria, and with the increase in the soil Cu levels, tolerant microorganisms were enriched but sensitive taxa were suppressed. Mycorrhizal fungi increased in the low Cu (Cu 108) treatment, while pathogenic fungi were boosted in the high Cu (Cu 178, Cu 318, and Cu 388) treatment.

### 4.3. The Interaction between Microorganisms and Soil Chemistry Properties and Plant Biomass and Nutrient Concentrations

Cu is essential for plant growth, but excess Cu can be toxic and inhibit plant growth and nutrients absorption [12]. Cu toxicity in plants is typically characterized by a significant inhibition of root growth, and the plant biomass serves as a sensitive indicator of the plant’s response to varying Cu levels [79]. In this study, we also confirmed that the plant biomass increased under the low Cu treatment (Cu 178), while the high Cu treatments (Cu 388) led to a reduction in the plant biomass (Figure 1). One possible explanation for this phenomenon is that an appropriate increase in Cu concentration can enhance root cellular respiration and protein synthesis, thus promoting root growth [80]. However, a high concentration of Cu ions can damage the root outer layer, resulting in a decrease in root hair, ultimately leading to root death [81]. This showed that there was a significant negative correlation between the plant biomass and soil Cu (Figure 6C). Moreover, the plant biomass was also mainly positively correlated with the soil AN and AP in the RDA (Figure 6D), because N and P are essential nutritional elements that can dramatically boost plant biomass and growth [82]. What is more, the plant N and P concentrations were mainly positively correlated with the soil AP, AN, and ACP (Figure 6D), which was because the improved nutrient contents in the soil could enhance the nutrient uptake and benefit plant growth [83].

Plant growth is not only influenced by soil nutrients but also widely related to soil microbes, especially fungi. The relationship is complex, and the outcomes vary depending on the plant [84]. The diversity of fungi and bacteria in agroecosystems plays a crucial role in promoting plant growth and nutrients cycling, such as soil N and P [85]. In this study, the Procrustes analysis revealed the correlation between microorganism and citrus biomass and nutrient (Figure 6A,B), and the low values of M2 (bacteria: 0.2755; fungi: 0.3827) and *p* (bacteria: 0.001; fungi: 0.001) indicated significant congruence relationships between the soil microbial community and citrus biomass and nutrient concentrations (Figure 6A,B). A previous report showed that *Terrabacter* has been found to have the ability to promote biological P removal [86]. Additionally, certain unidentified species of *Rhodopseudomonas* have been observed to produce growth-promoting substances like IAA and ALA, thereby facilitating the germination of tomato seeds and promoting overall plant growth [87,88].

A co-occurrence network analysis was performed to investigate the correlation between the bacterial community and fungal community with citrus biomass and nutrient concentrations under different soil Cu levels (Figure 7). *Actinomycetes* were the primary bacterial community involved in the network analysis and were closely associated with the plant nutrient concentrations. This result can be explained by the fact that dominant microorganisms often have a relatively high abundance in the soil and play a significant role in regulating ecological functions [89]. In addition, *Actinobacteriota* and *Proteobacteria* were mainly negatively correlated with the plant Cu concentration with the rise in the soil Cu levels. Moreover, *Actinobacteriota* were primarily positively correlated with the plant biomass and the concentrations of the N, P, and K as the soil Cu levels increased (Figure 7). However, *Proteobacteria* was mainly positively correlated with the plant N concentration at the low Cu treatment but showed a negative correlation with the plant N and K concentrations and a positive correlation with the plant P at the high Cu treatment. Certain bacteria that promote plant growth have a significant impact on the assimilation and utilization of essential nutrients in plants, including N fixation, P dissolution, and the production of iron carriers [90,91]. Furthermore, these bacteria contribute to pest control; enhance the resistance to environmental stressors such as high temperatures, high salt levels, drought, and heavy metals; and facilitate plant growth, development, and immunity by producing plant hormones [92,93].

*Ascomycota* and *Basidiomycota* were the primary fungal communities involved in the network analysis (Figure 7D–F). *Ascomycota* exhibited a remarkable positive correlation with the plant Cu concentration at different soil Cu levels. Generally, *Ascomycota* mainly had a significant negative correlation with the plant biomass and N, P, and K concentrations when the soil Cu levels increased. *Basidiomycota* were mainly positively correlated with the plant Cu and P concentrations. Both *Ascomycota* and *Basidiomycota* are predominantly saprophytic fungi, serving as key decomposers of nutrients in rhizosphere soil and commonly dominating fungal communities [94]. Specifically, *Ascomycota* primarily decomposes refractory organic matter, whereas *Basidiomycota* are decomposers of lignin and cellulose, forming ectomycorrhizal associations with plants [95,96]. Interestingly, *Glomeromycota* (Arbuscular mycorrhizal, AM) displayed a positive correlation with the plant P uptake (Figure 7D). However, the role of *Glomeromycota* in the plant biomass and nutrient concentrations weakened with the increasing soil Cu levels (Figure 7E,F), consistent with the decrease in the relative abundance of mycorrhizal fungi at the higher Cu treatment (Figure 5B). AM fungi play a vital role in plant nutrients uptake [97], heavy metal tolerance, accumulation, and transportation from roots to aboveground plant structures [98,99]. Consequently, the decrease in plant P concentration might be attributed to the reduction in AM fungi abundance under the high Cu treatment (Figure 1). What is more, *Mortierellomycota*, which contains pathogenic fungi, was mainly negatively correlated with the plant K concentration (Figure 7F). Thus, the soil Cu levels affected the composition and structure of the microbe communities, altered the soil environmental conditions, and the plant biomass and nutrient concentrations had a close relationship with the soil microbe communities and chemistry properties.

## 5. Conclusions

In this study, we confirmed that the increasing soil Cu levels altered the structure of the soil microbial communities, soil nutrient activity, and citrus biomass and nutrient concentration. When the soil Cu levels increased, the soil TCu, ACu, SOM, AK, and pH increased, while the soil AP and AN decreased. Moreover, the soil extracellular enzymes activities related to C and P metabolism decreased, while the enzymes related to N metabolism increased, and the expression of soil genes involved in C, N, and P cycling was regulated. The citrus biomass was mainly positively correlated with the soil AN and AP; the plant N and P were mainly positively correlated with the soil AP, AN, and acid phosphatase (ACP); and the plant K was mainly negatively related with soil β−glucosidase (βG) and positively related with the soil fungal Shannon index. *Actinobacteriota* were positively correlated with the plant biomass and plant N, P, and K and were negatively correlated with the plant Cu. *Ascomycota* was positively related to the plant Cu but negatively correlated with the plant biomass and plant N, P, and K. Notably, arbuscular mycorrhizal fungi (*Glomeromycota*) were positive related with the plant P below soil Cu 108 mg kg^−1^, and the pathogenic fungi (*Mortierella*) were negatively correlated with the plant K above soil Cu 178 mg kg^−1^. Thereby, the citrus biomass and nutrient concentration have a close and complex relationship with the soil nutrient activity and microbial community structure with the increasing soil Cu levels. These findings provided a new perspective on soil Cu nutrient management and the healthy development of the citrus industry.

## Figures and Tables

**Table 1:** Characteristics of the soil chemical properties in *Citrus reticulata* cv. Shatangju rhizosphere under different soil Cu levels.

Treatments	TCu (mg kg^−1^)	ACu (mg kg^−1^)	SOM (g kg^−1^)	pH	AN (mg kg^−1^)	AP (mg kg^−1^)	AK (mg kg^−1^)
Cu 38	33.18 ± 0.39 e	0.47 ± 0.04 e	16.77 ± 0.95 d	6.68 ± 0.10 a	70.74 ± 7.62 a	39.12 ± 3.70 a	493.13 ± 18.73 b
Cu 108	69.85 ± 2.70 d	25.40 ± 1.30 d	17.91 ± 0.25 cd	6.58 ± 0.02 b	64.89 ± 4.40 ab	36.66 ± 5.67 a	550.38 ± 26.68 a
Cu 178	103.30 ± 4.59 c	42.16 ± 3.11 c	19.69 ± 0.37 ab	6.57 ± 0.03 b	72.55 ± 1.32 a	33.94 ± 2.47 a	532.82 ± 3.25 a
Cu 318	167.12 ± 5.81 b	87.79 ± 2.69 b	20.48 ± 0.92 a	6.57 ± 0.05 b	59.87 ± 1.38 b	14.45 ± 0.74 b	566.00 ± 4.55 a
Cu 388	201.27 ± 13.76 a	115.65 ± 10.52 a	18.63 ± 0.71 bc	6.51 ± 0.03 b	49.96 ± 2.54 c	11.99 ± 0.74 b	550.06 ± 20.50 a

Note: Different lowercase letters indicate significant differences among the treatments by Duncan-test (*p* < 0.05, *n* = 3).

**Table 2:** Characteristics of the soil extracellular enzyme activities in *Citrus reticulata* cv. Shatangju rhizosphere under different soil Cu levels.

Treatments	FRO /U g^−1^	αG /nmol (g h)^−1^	βG /nmol (g h)^−1^	CBH /nmol (g h)^−1^	βX /nmol (g h)^−1^	NAG /nmo (g h)^−1^	ACP /nmol (g h)^−1^
Cu 38	1.85 ± 0.08 e	6.37 ± 0.14 a	5.20 ± 0.06 a	1.16 ± 0.04 b	1.05 ± 0.04 a	2.06 ± 0.12 d	11.31 ± 0.26 a
Cu 108	4.84 ± 0.09 c	5.04 ± 0.02 b	4.09 ± 0.06 b	1.05 ± 0.05 cd	1.08 ± 0.04 a	3.97 ± 0.11 b	11.38 ± 0.23 a
Cu 178	5.85 ± 0.06 b	3.63 ± 0.04 c	4.69 ± 0.12 a	1.06 ± 0.01 c	1.06 ± 0.02 a	4.19 ± 0.11 a	10.80 ± 0.26 a
Cu 318	6.21 ± 0.13 a	1.11 ± 0.09 d	5.11 ± 0.08 a	1.57 ± 0.06 a	0.81 ± 0.02 c	3.2 ± 0.04 c	5.49 ± 0.13 c
Cu 388	3.44 ± 0.17 d	1.10 ± 0.07 d	3.96 ± 0.60 b	0.98 ± 0.04 d	0.88 ± 0.01 b	2.24 ± 0.09 d	6.54 ± 0.55 b

Note: Different lowercase letters indicate significant differences among the treatments by Duncan-test (*p* < 0.05, *n* = 3).

## Data Availability

Data are contained within the article and Supplementary Materials.

## Supplementary Materials

The following supporting information can be downloaded at https://www.mdpi.com/article/10.3390/plants13172344/s1: Figure S1. The vector characteristics of the soil extracellular enzyme activities under different soil Cu levels. (A,B) The vector length and vector angle, respectively. Cu 38, Cu 108, Cu 178, Cu 318, and Cu 388 indicate soil with 38 mg kg^−1^, 108 mg kg^−1^, 178 mg kg^−1^, 318 mg kg^−1^, and 388 mg kg^−1^ Cu, respectively. C/(C + P): (βG + CBH + βX + αG)/(βG + CBH + βX + αG + ACP); C/(C + N): (βG + CBH + βX + αG)/(βG + CBH + βX + αG + NAG). Vector length = SQRT (X^2^ + Y^2^). Vector angle = DEGREES (ATAN2 (X, Y)). X = C/(C + P); Y = C/(C + N). Different lowercase letters indicate significant differences according to Duncan’s test (*p* < 0.05, n = 3). Figure S2. Rarefaction curves of bacteria (A) and fungi (B) species of root microbiota in *Citrus reticulata* cv. Shatangju rhizosphere under different soil Cu levels. Cu 38, Cu 108, Cu 178, Cu 318, and Cu 388 indicate soil with 38 mg kg^−1^, 108 mg kg^−1^, 178 mg kg^−1^, 318 mg kg^−1^, and 388 mg kg^−1^ Cu, respectively. Figure S3. Characteristics of differential microbiota compositions with the abundant genus (Top 20) (A: bacteria, and B: fungi) in *Citrus reticulata* cv. Shatangju rhizosphere under different soil Cu levels. CK indicated soil with 38 mg kg^−1^ Cu; Low Cu indicated soil with 108 mg kg^−1^ Cu; and High Cu indicated soil with 178 mg kg^−1^, 318 mg kg^−1^, and 388 mg kg^−1^ Cu, respectively. Asterisks denoted significant differences (* *p* < 0.05, ** *p* < 0.01). Figure S4. Differential analysis of carbon (A), nitrogen (B), and phosphorus (C) metabolism functional genes in *Citrus reticulata* cv. Shatangju rhizosphere under different soil Cu levels. Red represented the genus in high abundance, while blue represented low abundance. CK indicated soil with 38 mg kg^−1^ Cu; Low Cu indicated soil with 108 mg kg^−1^ Cu; and High Cu indicated soil with 178 mg kg^−1^, 318 mg kg^−1^, and 388 mg kg^−1^ Cu, respectively.

## References

[1] Mamma D., Christakopoulos P. (2014). Biotransformation of citrus by-products into value added products. Waste Biomass Valori..

[2] Zema D., Calabrò P., Folino A., Tamburino V., Zappia G., Zimbone S. (2018). Valorisation of citrus processing waste: A review. Waste Manag..

[3] Mahato N., Sharma K., Sinha M., Baral E.R., Koteswararao R., Dhyani A., Cho M.H., Cho S. (2020). Bio-sorbents, industrially important chemicals and novel materials from citrus processing waste as a sustainable and renewable bioresource: A review. J. Adv. Res..

[4] Zou Z., Xi W., Hu Y., Nie C., Zhou Z. (2016). Antioxidant activity of Citrus fruits. Food Chem..

[5] Yao T.S., Zhou Y., Zhou C.Y. (2016). Advances in Copper Resistant Mechanisms and Control Methods of Citrus Canker. Acta Hortic. Sin..

[6] Girotto E., Ceretta C.A., Brunetto G., Miotto A., Tiecher T.L., De Conti L., Lourenzi C.R., Lorensini F., Gubiani P.I., Da Silva L.S. (2014). Copper availability assessment of Cu contaminated vineyard soils using black oat cultivation and chemical extractants. Environ. Monit. Assess..

[7] Fan J., He Z., Ma L.Q., Stoffella P.J. (2011). Accumulation and availability of copper in citrus grove soils as affected by fungicide application. J. Soils Sediments.

[8] Ying J.G., Liu X.H., Li J.B., Wu Q., Peng S.A., Jiang C.C. (2016). Analysis of Cu and Mn contents in the soil and leaves of citrus orchards in Nanfeng and Quzhou. South China Fruits.

[9] Zhao Y.B., Han J., Yang G.B., Long L.Z., Tan Z.H., Li X.X., Zhou W.J., Deng Z.N., Ma X.F. (2020). Analysis of soil and leaf and fruit mineral elements in the main sweet orange producing areas in Hunan Province. South China Fruits.

[10] Jiang Y.N., Fu H.M., Liu B.H., Liu S.Q., Tang X.S., Zhai J., Deng C.L. (2020). Research on soil and leaf nutrient status in red soil navel orange orchard. South Horticult..

[11] Mo X., Chen C., Riaz M., Moussa M.G., Chen X., Wu S., Tan Q., Sun X., Zhao X., Shi L. (2022). Fruit Characteristics of Citrus Trees Grown under Different Soil Cu Levels. Plants.

[12] Hippler F.W., Cipriano D.O., Boaretto R.M., Quaggio J.A., Gaziola S.A., Azevedo R.A., Mattos-Jr D. (2016). Citrus rootstocks regulate the nutritional status and antioxidant system of trees under copper stress. Environ. Exp. Bot..

[13] Saleh T.A. (2020). Nanomaterials: Classification, properties, and environmental toxicities. Environ. Technol. Innov..

[14] Xia H., Riaz M., Tang X., Yan L., El-Desouki Z., Li Y., Wang X., Jiang C. (2023). Insight into mechanisms of biochar-fertilizer induced of microbial community and microbiology of nitrogen cycle in acidic soil. J. Environ. Manag..

[15] Han G.Q., Wang B., Chen G.Q., Wang H.X., Zhang H.B., Zhang X.J., Xiong Z.T. (2010). Effects of heavy metal combined pollution on soil microbial indicators and soil enzymatic activity. J. Soil Water Conserv..

[16] Dai J., Becquer T., Rouiller J.H., Reversat G., Bernhard-Reversat F., Lavelle P. (2004). Influence of heavy metals on C and N mineralisation and microbial biomass in Zn-, Pb-, Cu-, and Cd-contaminated soils. Appl. Soil Ecol..

[17] Cui J.L., Zhao Y.P., Chan T.S., Zhang L.L., Tsang D.C., Li X.D. (2020). Spatial distribution and molecular speciation of copper in indigenous plants from contaminated mine sites: Implication for phytostabilization. J. Hazard Mater..

[18] Chung H., Kim M.J., Ko K., Kim J.H., Kwon H.A., Hong I., Park N., Lee S.W., Kim W. (2015). Effects of graphene oxides on soil enzyme activity and microbial biomass. Sci. Total Environ..

[19] Deforest J.L., Moorhead D.L. (2020). Effects of elevated pH and phosphorus fertilizer on soil C, N and P enzyme stoichiometry in an acidic mixed mesophytic deciduous forest. Soil Biol. Biochem..

[20] Wu J.X., Li S.Y., Han G.D. (2024). Study on soil carbon, nitrogen, phosphorus acquisition enzyme activity, and microbial entropy in Stipa breviflora desert grassland of Inner Mongolia. Pratacultural Sci..

[21] Allison S.D., Vitousek P.M. (2005). Responses of extracellular enzymes to simple and complex nutrient inputs. Soil. Biol. Biochem..

[22] Upadhyay L.S.B. (2012). Urease inhibitors: A review. Indian J. Biotechnol..

[23] Wang J., Zhao Y., Dai T., Fan X., Liu Y., Tang B. (2014). Influence of Cu and Cd Pollution on Activeness of Urease in Soil. Environ. Sci. Manag..

[24] Mounissamy V.C., Kundu S., Selladurai R., Saha J.K., Biswas A.K., Adhikari T., Patra A.K. (2017). Effect of soil amendments on microbial resilience capacity of acid soil under copper stress. Bull. Environ. Contam. Toxcol..

[25] Moorhead D.L., Sinsabaugh R.L., Hill B.H., Weintraub M.N. (2016). Vector analysis of ecoenzyme activities reveal constraints on coupled C, N and P dynamic. Soil Biol. Biochem..

[26] Cui Y.X., Fang L.C., Deng L., Guo X.B., Han F., Ju W.L., Wang X., Chen H.S., Tan W.F., Zhang X.C. (2019). Patterns of soil microbial nutrient limitations and their roles in the variation of soil organic carbon across a precipitation gradient in an arid and semi-arid region. Sci. Total Environ..

[27] Zhang C., Nie S., Liang J., Zeng G., Wu H., Hua S., Liu J., Yuan Y., Xiao H., Deng L. (2016). Effects of heavy metals and soil physicochemical properties on wetland soil microbial biomass and bacterial community structure. Sci. Total Environ..

[28] Chen Y., Ding Q., Chao Y. (2018). Structural development and assembly patterns of the root-associated microbiomes during phytoremediation. Sci. Total Environ..

[29] Li Destri Nicosia M.G., Mosca S., Mercurio R., Schena L. (2015). Dieback of pinus nigra seedlings caused by a strain of trichoderma viride. Plant Dis..

[30] Zhou X., He Z., Liang Z., Stoffella P.J., Fan J., Yang Y., Powell C.A. (2011). Long-term use of copper-containing fungicide affects microbial properties of citrus grove soils. Soil Sci. Soc. Am. J..

[31] Schmidt R., Mitchell J., Scow K. (2019). Cover cropping and no-till increase diversity and symbiotroph: Saprotroph ratios of soil fungal communities. Soil Biol. Biochem..

[32] Wu S., Zhang C., Li M., Tan Q., Sun X., Pan Z., Deng X., Hu C. (2021). Effects of potassium on fruit soluble sugar and citrate accumulations in Cara Cara navel orange (*Citrus sinensis* L.. Osbeck). Sci. Hortic..

[33] Bao S.D. (2000). Soil and Agriculture Chemistry Analysis.

[34] Harrison S., Lepp N., Phipps D. (1979). Uptake of Copper by excised roots II. Copper desorption from the free space. Z Pflanzenphysiol..

[35] Moussa M.G., Sun X., Ismael M.A., Elyamine A.M., Rana M.S., Syaifudin M., Hu C. (2021). Molybdenum-induced effects on grain yield, macro–micro-nutrient uptake, and allocation in mo-inefficient winter wheat. J. Plant Growth Regul..

[36] DeForest J.L. (2009). The influence of time, storage temperature, and substrate age on potential soil enzyme activity in acidic forest soils using MUB-linked substrates and l-DOPA. Soil Biol. Biochem..

[37] Liao X., Chen C., Zhang J., Dai Y., Zhang X., Xie S. (2015). Operational performance, biomass and microbial community structure: Impacts of backwashing on drinking water biofilter. Environ. Sci. Pollut. Res..

[38] Adams R.I., Miletto M., Taylor J.W., Bruns T.D. (2013). Dispersal in microbes: Fungi in indoor air are dominated by outdoor air and show dispersal limitation at short distances. ISME J..

[39] Zhang M.Y., Riaz M., Liu B., Xia H., El-desouki Z., Jiang C.C. (2020). Two-year study of bio-char: Achieving excellent capability of potassium supply via alter clay mineral composition and potassium-dissolving bacteria activity. Sci. Total Environ..

[40] Chen S., Zhou Y., Chen Y. (2018). FASTP: An ultra-fast all-in-one FASTQ preprocessor. Bioinformatics.

[41] Magoč T., Salzberg S.L. (2011). FLASH: Fast length adjustment of short reads to improve genome assemblies. Bioinformatics.

[42] Edgar R.C. (2013). UPARSE: Highly accurate OTU sequences from microbial amplicon reads. Nat. Methods.

[43] Wang Q. (2007). Naive Bayesian classifier for rapid assignment of rRNA sequences into the new bacterial taxonomy. Appl. Environ. Microbiol..

[44] Schloss P.D., Westcott S.L., Ryabin T. (2009). Introducing mothur: Open-Source, Platform-Independent, Community-Supported Software for Describing and Comparing Microbial Communities. Appl. Environ. Microb..

[45] Pu S.Y., Wang Y., Chen W.Y. (2020). Review on the mechanism of plant rhizosphere soil enzyme response to heavy metal pollution. Asian J. Ecotox..

[46] Zhang M.K., Wang L.P. (2007). Impact of heavy metals pollution on soil organic matter accumulation. Chin. J. Appl. Ecol..

[47] Wyszkowska J., Borowik A., Kucharski M., Kucharski J. (2013). Effect of cadmium, copper and zinc on plants, soil microorganisms and soil enzymes. J. Elementol..

[48] Ali N., Dashti N., Al-Mailem D., Eliyas M., Radwan S. (2012). Indigenous soil bacteria with the combined potential for hydrocarbon consumption and heavy metal resistance. Environ. Sci. Pollut. Res..

[49] Waring B.G., Weintraub S.R., Sinsabaugh R.L. (2014). Ecoenzymatic stoichiometry of microbial nutrient acquisition in tropical soils. Biogeochemistry.

[50] Stone L.F. (2006). Physical, chemical, and biological changes in the rhizosphere and nutrient availability. J. Plant Nutr..

[51] White P.J., Broadley M.R. (2009). Biofortification of crops with seven mineral elements often lacking in human diets-iron, zinc, copper, calcium, magnesium, selenium and iodine. New Phytol..

[52] Burkhead J.L., Gogolin Reynolds K.A., Abdel-Ghany S.E., Cohu C.M., Pilon M. (2009). Copper homeostasis. New Phytol..

[53] Frąc M., Oszust K., Lipiec J. (2012). Community level physiological profiles (CLPP), characterization and microbial activity of soil amended with dairy sewage sludge. Sensors.

[54] Azarbad H., Niklińska M., Van Gestel C.A., Van Straalen N.M., Röling W.F., Laskowski R. (2013). Microbial community structure and functioning along metal pollution gradients. Environ. Toxicol. Chem..

[55] Ge Y., Xu M.M., Xu S.H., Xu Y. (2021). Effects of Copper Pollution on Microbial Communities in Wheat Root System. Environ. Sci..

[56] Krumova E., Andreyinski N., Abrashev R., Stoyancheva G., Kostadinova N., Miteva-Staleva J., Dishlijska V., Spasova B., Angelova M. (2021). Comparison of the oxidative stress response of two aspergillus fumigatus strains isolated from polluted soils against combined heavy metal toxicity. Geomicrobiol. J..

[57] Fernandez-Calvio D., Bååth E. (2016). Interaction between pH and Cu toxicity on fungal and bacterial performance in soil. Soil Biol. Biochem..

[58] Wu S., Wu K., Shi L., Sun X., Tan Q., Hu C. (2023). Recruitment of specific microbes through exudates affects cadmium activation and accumulation in Brassica napus. J. Hazard. Mater..

[59] Lv Z., Rønn R., Liao H., Rensing C., Chen W., Huang Q., Hao X. (2023). Soil aggregates affect the legacy effect of copper pollution on the microbial communities. Soil Biol.Biochem..

[60] Sun L., Zhang Y., He L. (2010). Genetic diversity and characterization of heavy metal-resistant endophytic bacteria from two copper-tolerant plant species on copper mine wasteland. Bioresour. Technol..

[61] Guo D.C., Fan Z.Z., Lu S.Y., Ma Y.J., Nie X.H., Tong F.P., Peng X.W. (2019). Changes in rhizosphere bacterial communities during remediation of heavy metal accumulating plants around the Xikuangshan mine in southern China. Sci. Rep..

[62] Camacho-Chab J.C., Castañeda-Chávez M.D.R., Chan-Bacab M.J. (2018). Biosorption of cadmium by non-toxic extracellular polymeric substances (EPS) synthesized by bacteria from marine intertidal biofilms. Int. J. Environ. Res. Public Health.

[63] Hayat R., Ali S., Amara U. (2010). Soil beneficial bacteria and their role in plant growth promotion: A review. Ann. Microbiol..

[64] Pan F.S., Meng Q., Wang Q., Luo S., Chen B., Khan K.Y., Yang X.E., Feng Y. (2016). Endophytic bacterium Sphingomonas SaMR12 promotes cadmium accumulation by increasing glutathione biosynthesis in Sedum alfredii Hance. Chemosphere.

[65] Granja-Travez R.S., Bugg T.D.H. (2018). Characterization of multicopper oxidase CopA from Pseudomonas putida KT2440 and Pseudomonas fluorescens Pf-5: Involvement in bacterial lignin oxidation. Arch. Biochem. Biophys..

[66] Zhao Y., Gao J., Wang Z. (2021). Responses of bacterial communities and resistance genes on microplastics to antibiotics and heavy metals in sewage environment. J. Hazard. Mater..

[67] Singh B.K., Quince C., Macdonald C.A., Khachane A., Thomas N., Abu Al-Soud W., Sorensen S.J., He Z., White D., Sinclair A. (2014). Loss of microbial diversity in soils is coincident with reductions in some specialized functions. Environ. Microbiol..

[68] Pereira S.I.A., Lima A.I.G., Figueira E.M.D.A.P. (2006). Screening possible mechanisms mediating cadmium resistance in Rhizobium leguminosarum bv. viciae isolated from contaminated Portuguese soils. Microb. Ecol..

[69] Segata N., Izard J., Waldron L., Gevers D., Miropolsky L., Garrett W.S., Huttenhower C. (2011). Metagenomic biomarker discovery and explanation. Genome Biol..

[70] Gowthami S., Thirumarimurugan M., Sivakumar V.M., Sukanya K. (2017). Impending heavy metal tolerance of fungus isolated from dye industrial sludge. Int. J. Mater. Prod. Technol..

[71] Cai Z., Du W., Zhang Z., Guan L., Zeng Q., Chai Y., Dai C., Lu L. (2018). The aspergillus fumigatus transcription factor AceA is involved not only in Cu but also in Zn detoxification through regulating transporters CrpA and ZrcA. Cell. Microbiol..

[72] Lacerda E.C.M., dos Passos Galluzzi Baltazar M., dos Reis T.A., do Nascimento C.A.O., Côrrea B., Gimenes L.J. (2019). Copper biosorption from an aqueous solution by the dead biomass of *penicillium* ochrochloron. Environ. Monit. Assess..

[73] Zhang X., Fu G., Xing S., Fu W., Liu X., Wu H., Chen B. (2022). Structure and diversity of fungal communities in long-term copper-contaminated agricultural soil. Sci. Total Environ..

[74] Awasthi A., Singh M., Soni S.K., Singh R., Kalra A. (2014). Biodiversity acts as insurance of productivity of bacterial communities under abiotic perturbations. ISME J..

[75] Han J.J., Shen X., Yang F., Wang F., Qin C.Y., Zou D.Y., Hu Q.Y., Lin J.X., Wang J.H. (2023). Research progress on the mechanism of Arbuscular Mycorrhizal Fungi (AMF) Mediated Mineral Elements Uptake by Plants. Acta Agrestia Sin..

[76] Yim B., Smalla K., Winkelmann T. (2013). Evaluation of apple replant problems based on different soil disinfection treatments-links to soil microbial community structure?. Plant Soil.

[77] Emmett B., Nelson E.B., Kessler A., Bauerle T.L. (2014). Fine-root system development and susceptibility to pathogen colonization. Planta.

[78] Weiβ S., Bartsch M., Winkelmann T. (2017). Transcriptomic analysis of molecular responses in Malus domestica ‘M26’ roots affected by apple replant disease. Plant Mol. Biol..

[79] Daud M., Ali S., Variath M., Zhu S. (2013). Differential physiological, ultramorphological and metabolic responses of cotton cultivars under cadmium stress. Chemosphere.

[80] Zeng Q., Ling Q., Wu J., Yang Z., Liu R., Qi Y. (2019). Excess copper-induced changes in antioxidative enzyme activity, mineral nutrient uptake and translocation in sugarcane seedlings. Bull. Environ. Contam. Toxcol..

[81] Miotto A., Ceretta C.A., Brunetto G., Nicoloso F.T., Girotto E., Farias J.G., Tiecher T.L., De Conti L., Trentin G. (2014). Copper uptake, accumulation and physiological changes in adult grapevines in response to excess copper in soil. Plant Soil.

[82] Cheng Z.Y., Shi J.C., He Y., Chen Y.X., Wang Y.J., Yang X.L., Wang T.Y., Wu L.S., Xu J.M. (2023). Enhanced soil function and health by soybean root microbial communities during in situ remediation of Cd-contaminated soil with the application of soil amendments. mSystems.

[83] Mohammad G., Petr K., Reinhard W.N., Marek K., Elnaz A., Moudrý J., Ladislav M. (2022). Preliminary Findings on Cadmium Bioaccumulation and Photosynthesis in Rice (*Oryza sativa* L.) and Maize (*Zea mays* L.) Using Biochar Made from C3- and C4-Originated Straw. Plants.

[84] Zeilinger S., Gupta V.K., Dahms T.E.S., Silva R.N., Singh H.B., Upadhyay R.S., Gomes E.V., Tsui C.K.-M., Nayak C.S., van der Meer J.R. (2016). Friends or foes? Emerging insights from fungal interactions with plants. FEMS Microbiol. Rev..

[85] Bennett A.E., Classen A.T. (2020). Climate change influences mycorrhizal fungal-plant interactions, but conclusions are limited by geographical study bias. Ecology.

[86] Seviour R.J., Mino T., Onuki M. (2003). The microbiology of biological phosphorus removal in activated sludge systems. FEMS Microbiol. Rev..

[87] Koh R.H., Song H.G. (2007). Effects of application of Rhodopseudomonas sp. on seed germination and growth of tomato under axenic conditions. J. Microbiol. Biotechnol..

[88] Lee K.H., Koh R.H., Song H.G. (2008). Enhancement of growth and yield of tomato by Rhodopseudomonas sp. under greenhouse conditions. J. Microbiol..

[89] Wang Y., Cheng D.H., Tan W.B., Yu H., Xi B.D., Jiang Y.H., Dang Q.L. (2020). Different Responses of Soil Microbial Community Structure to Irrigation with Treated Wastewater from Domestic and Industrial Sources. Environ. Sci..

[90] Gray E.J., Smith D.L. (2005). Intracellular and extracellular PGPR: Commonalities and distinctions in the plant-bacterium signaling processes. Soil Biol. Biochem..

[91] Lakshmanan V., Kitto S.L., Caplan J.L. (2012). Microbe-associated molecular patterns-triggered root responses mediate beneficial rhizobacterial recruitment in Arabidopsis. Plant Physiol..

[92] Xu Y.X., Wang G.H., Jin J. (2009). Bacterial communities in soybean rhizosphere in response to soil type, soybean genotype, and their growth stage. Soil Biol. Biochem..

[93] Finkel O.M., Castrillo G., Paredes S.H. (2017). Understanding and exploiting plant beneficial microbes. Curr. Opin. Plant Biol..

[94] Jinbo X., Fei P., Huaibo S. (2014). Erratum to: Divergent responses of soil fungi functional groups to short-term warming. Microb. Ecol..

[95] Junsheng H., Bin H., Kaibin Q. (2016). Effects of phosphorus addition on soil microbial biomass and community composition in a subalpine spruce plantation. Eur. J. Soil Biol..

[96] Chen Y.Y., Xia W.Y., Zhao H., Zeng M. (2022). Effects of deep vertical rotary tillage on soil enzyme activity, microbial community structure, and functional diversity of cultivated land. Acta Ecol. Sin..

[97] Yao Q., Li X.L., Ai W.D., Christie P. (2003). Bi-directional transfer of phosphorus between red clover and perennial ryegrass via arbuscular mycorrhizal hyphal links. Eur. J. Soil Biol..

[98] Wu S., Zhang X., Sun Y., Wu Z., Li T., Hu Y., Su D., Lv J., Li G., Zhang Z. (2015). Transformation and immobilization of chromium by arbuscular mycorrhizal fungi as revealed by SEM-EDS, TEM-EDS, and XAFS. Environ. Sci. Technol..

[99] Li J., Sun Y., Jiang X., Chen B., Zhang X. (2018). Arbuscular mycorrhizal fungi alleviate arsenic toxicity to Medicago sativa by influencing arsenic speciation and partitioning. Ecotoxicol. Environ. Saf..

