# Supplementary material for: Characteristics of Rhizosphere Microbiome, Soil Chemical Properties, and Plant Biomass and Nutrients in Citrus reticulata cv. Shatangju Exposed to Increasing Soil Cu Levels
Source: Plants (Basel). 2024 Aug 23;13(17):2344. doi: 10.3390/plants13172344 (PMC11397084; doi:10.3390/plants13172344)
Supplement: Supplementary file 1 [file plants-13-02344-s001.zip › plants-3116424-supplementary.pdf]

Supplementary Figure

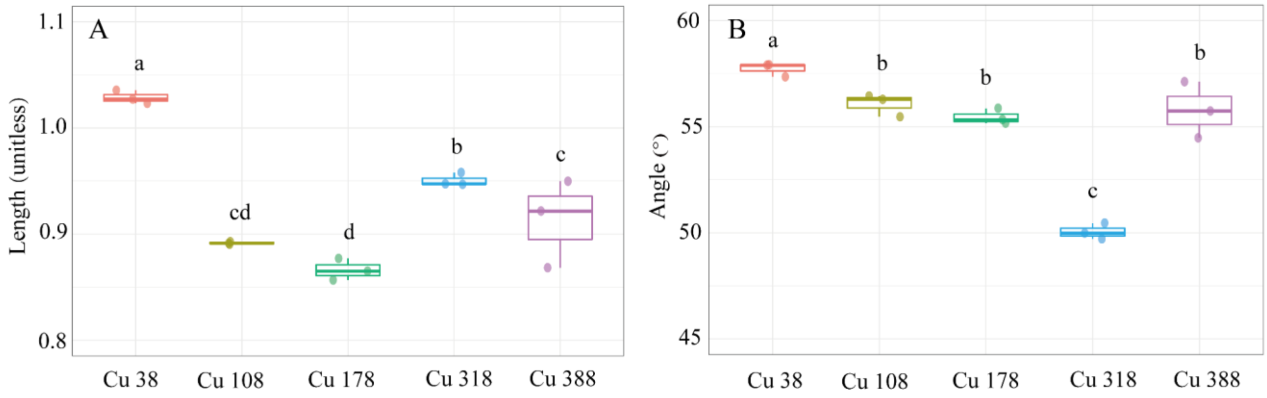

Fig. S1. The vector characteristics of soil extracellular enzyme activities under different soil Cu levels. A-B: represented the vector length and vector angle, respectively. The Cu 38, Cu 108, Cu 178, Cu 318 and Cu 388 indicate soil with 38 mg kg^{-1}, 108 mg kg^{-1}, 178 mg kg^{-1}, 318 mg kg^{-1}, 388 mg kg^{-1} Cu, respectively. C/(C+P): (βG + CBH + βX + αG) / (βG + CBH + βX + αG + ACP); C/(C+N): (βG + CBH + βX + αG) / (βG + CBH + βX + αG + NAG). Vector Length = SQRT ($X^2 + Y^2$). Vector Angle = DEGREES (ATAN2 (X, Y)). X=C/(C+P); Y=C/(C+N). Different lowercase letter indicated significant differences according to Duncan's test ($p < 0.05$, n=3).

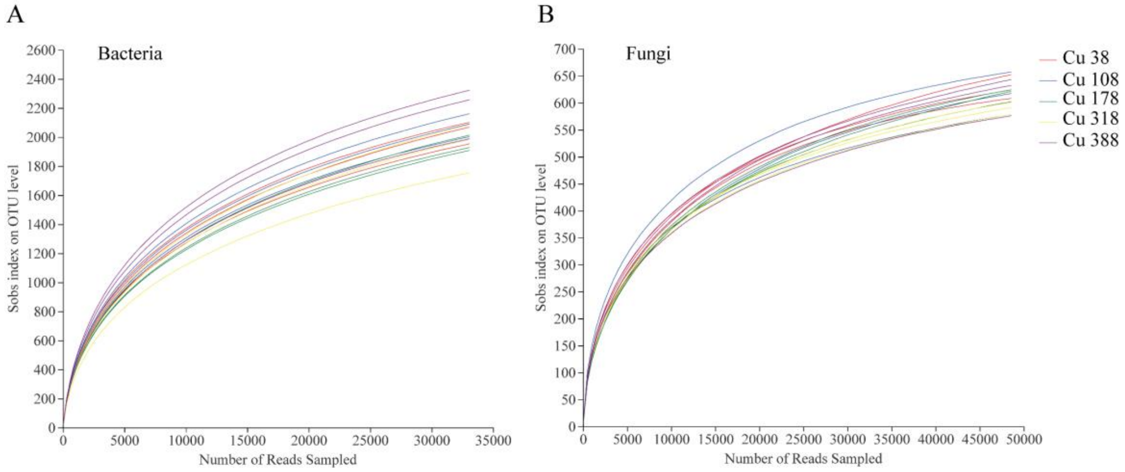

Fig. S2. Rarefaction curves of bacteria (A) and fungi (B) species of root microbiota in *Citrus reticulata* cv. Shatangju rhizosphere under different soil Cu levels. The Cu 38, Cu 108, Cu 178, Cu 318 and Cu 388 indicated soil with 38 mg kg^{-1}, 108 mg kg^{-1}, 178 mg kg^{-1}, 318 mg kg^{-1}, 388 mg kg^{-1} Cu, respectively.

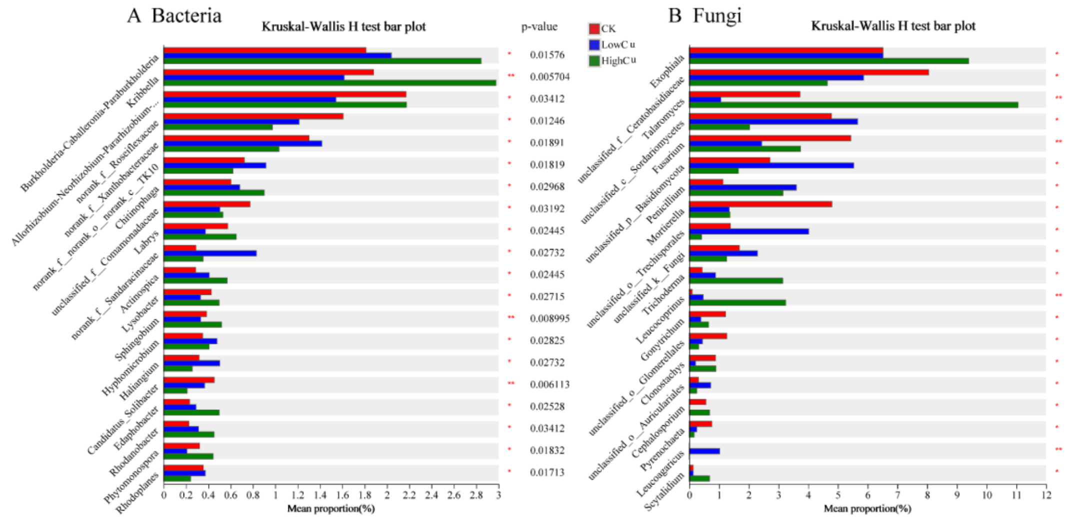

Fig. S3. Characteristics of differential microbiota compositions with the abundant genus (Top 20) (A: bacteria, B: fungi) in *Citrus reticulata* cv. Shatangju rhizosphere under different soil Cu levels. The CK indicated soil with 38 mg kg^{-1} Cu, Low Cu indicated soil with 108 mg kg^{-1} Cu, and High Cu indicated soil with 178 mg kg^{-1}, 318 mg kg^{-1}, 388 mg kg^{-1} Cu, respectively. Asterisks denoted significant differences (* $p < 0.05$, ** $p < 0.01$).

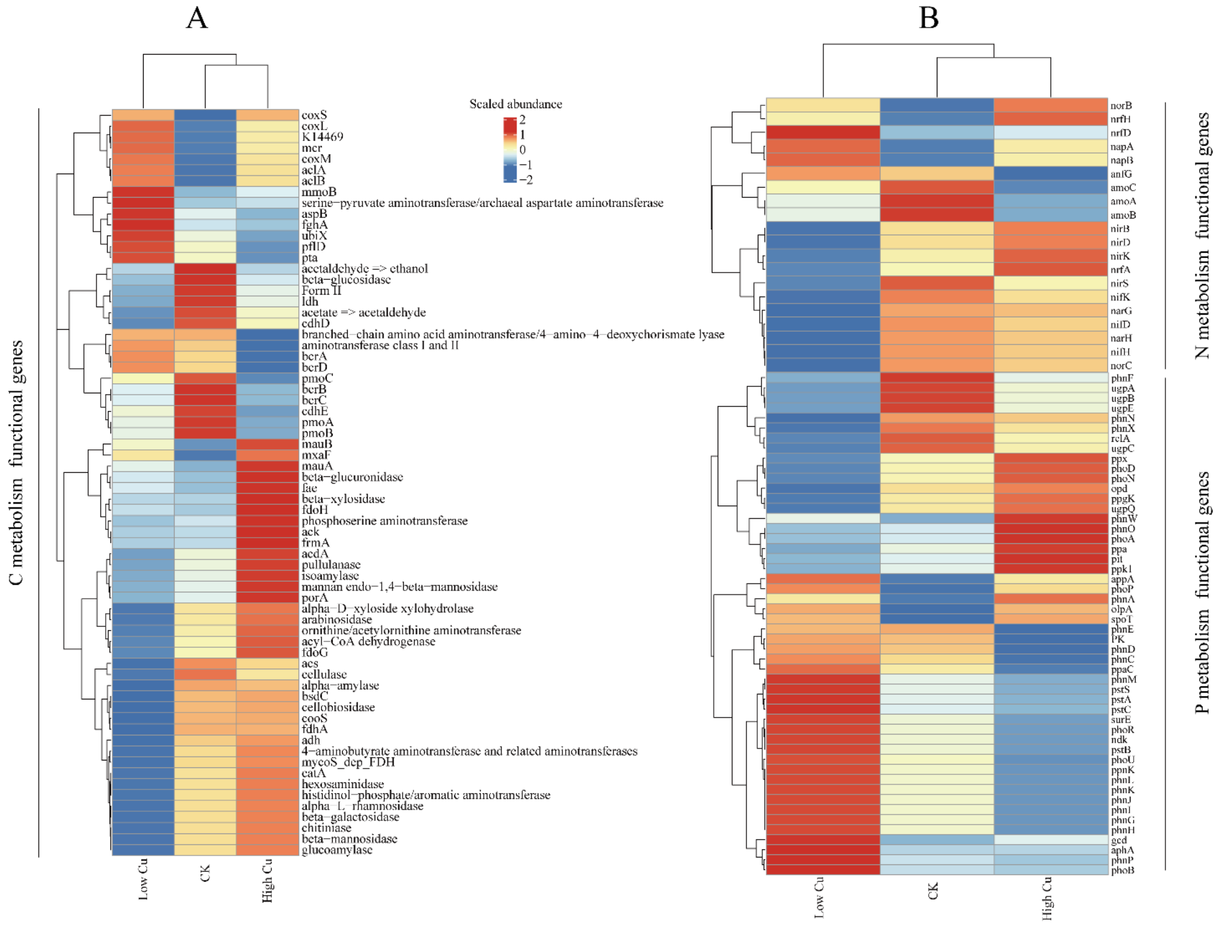

Fig. S4. Differential analysis of carbon (A), nitrogen (B) and phosphorus (B) metabolism functional genes in *Citrus reticulata* cv. Shatangju rhizosphere under different soil Cu levels. Red represented the genus in high abundance, while blue represented low abundance. The CK indicated soil with 38 mg kg^{-1} Cu, Low Cu indicated soil with 108 mg kg^{-1} Cu, and High Cu indicated soil with 178 mg kg^{-1}, 318 mg kg^{-1}, 388 mg kg^{-1} Cu, respectively.
